# MR imaging for detection of trampoline injuries in children

**DOI:** 10.1186/s12887-017-0791-2

**Published:** 2017-01-18

**Authors:** E. Hauth, H. Jaeger, P. Luckey, M. Beer

**Affiliations:** 1 Radiologische Praxis, Parkstraße 10, 89073 Ulm, Germany; 2 Department of Diagnostic and Interventional Radiology, University Hospital, Ulm, Germany

**Keywords:** MR imaging, Trampoline, Injuries, Fracture

## Abstract

**Background:**

The recreational use of trampolines is an increasingly popular activity among children and adolescents. Several studies reported about radiological findings in trampoline related injuries in children. The following publication presents our experience with MRI for detection of trampoline injuries in children.

**Methods:**

20 children (mean 9.2 years, range: 4–15 years) who had undergone an MRI study for detection of suspected trampoline injuries within one year were included. 9/20 (45%) children had a radiograph as the first imaging modality in conjunction with primary care. In 11/20 (55%) children MR imaging was performed as the first modality. MR imaging was performed on two 1.5 T scanners with 60 and 70 cm bore design respectively without sedation. In 9/20 (45%) children the injury mechanism was a collision with another child. 7/20 (35%) children experienced leg pain several hours to one day after using the trampoline without acute accident and 4/20 (20%) children described a fall from the trampoline to the ground.

**Results:**

All plain radiographs were performed in facilities outside the study centre and all were classified as having no pathological findings. In contrast, MR imaging detected injuries in 15/20 (75%) children. Lower extremity injuries were the most common findings, observed in 12/15 (80%) children. Amongst these, injuries of the ankle and foot were diagnosed in 7/15 (47%) patients. Fractures of the proximal tibial metaphysis were observed in 3/15 children. One child had developed a thoracic vertebral fracture. The two remaining children experienced injuries to the sacrum and a soft tissue injury of the thumb respectively. Seven children described clinical symptoms without an overt accident. Here, fractures of the proximal tibia were observed in 2 children, a hip joint effusion in another 2, and an injury of the ankle and foot in 1 child. There were no associated spinal cord injuries, no fracture dislocations, no vascular injuries and no head and neck injuries.

**Conclusions:**

In the majority of children referred for MR imaging with pain after trampoline MR imaging detects injuries. These injuries are often not visible on plain radiographs. Therefore we recommend a generous use of MR imaging in these children after initial negative plain radiography.

## Background

The recreational use of trampolines continues to be a popular activity among children and adolescents. An increasing number of trampoline injuries were reported in a published statement by the American Academy of Orthopaedic Surgeons in 2010 [1]. In this statement the Academy discouraged the use of trampolines in playgrounds and advised to further study the use of trampolines in supervised training and physical education settings.

Most trampoline injuries are manifestations of musculoskeletal injury mechanisms such as sprains, strains, contusions and other soft tissue injuries whereas younger children seem to be more prone to undergo bone injuries.

Several studies already described plain radiographic findings in trampoline related injuries in children [2–7]. The upper extremities show fractures, such as supracondylar humerus fractures or foream fractures due to direct impact on the ground or the frame of the trampoline. The lower extremities have a higher incidence of injuries. Axial forces to the ankle and knee joints explain growth arrest lines, widening of the growth plates, sclerosis of the metaphysis or Salter-Harris type fractures of the distal femur [5]. Fractures of the proximal tibia were the most common described injury [2–5].

The following publication presents our experience with MR imaging for detection of trampoline injuries in children.

## Methods

In 2014, we identified 20 children in whom MR imaging was performed for suspected trampoline injuries. The indication for MR imaging was done by the referring physician. Twelve girls and 8 boys with an age range from 4 to 15 years (mean 9.2 years) were examined. Five of these were six years or less of age. All injuries had occurred in the summer months from April to September 2014.

In 9/20 (45%) children the cause of injury was a collision with another child. 7/20 (35%) children developed leg pain several hours to one day after using the trampoline without overt accident and 4/20 (20%) children described a fall from the trampoline to the ground.

11/20 (55%) children had used a trampoline without a net and 9/20 (45%) children a net-secured device. Plain radiographs in two views were performed in institutions outside the study centre in 9/20 (45%) children. All radiographs were classified as normal without fracture signs. In 11/20 (55%) children MR imaging was the first imaging modality. MR imaging was performed after informed consent was obtained from the parents.

MR imaging was performed on a 1.5 T scanner (Magnetom Aera®; Siemens, Erlangen, Germany) with a 70 cm open bore design and a system length of 145 cm and on a 1.5 T scanner (Magnetom Symphony®, Siemens, Erlangen, Germany) with a bore diameter of 60 cm and a system length of 160 cm.

MR imaging of the thoracic and lumbar spine included a sagittal and coronal T2-weighted turbo inversion recovery magnitude (TIRM)- sequence for diagnosis of bone marrow oedema, sagittal T2-weighted turbo spin echo (TSE) and T1-weighted TSE-sequences for optimal diagnosis of fracture lines and vertebral endplate depression as well as a transverse T2-weighted TSE-sequence for optimal imaging of the spinal canal.

For MR imaging of the knee, ankle/foot and thumb we utilized proton (PD)-weighted sequences in coronal, sagittal and transverse planes for diagnosis of bone marrow oedema and a sagittal T1-weighted (TSE) sequence for optimal detection of fracture lines.

No intravenous contrast was administered and no sedation was performed. In 8/20 (40%) children a parent stayed in the scanner room during the acquisition to watch and if necessary talk to the child. The mean scan time was 15.7 min (Range: 10–32 min). No other imaging modality was employed after the MRI scan was obtained.

## Results

MR imaging of the following regions was performed: Thoracic spine in 4/20 (20%), lumbar spine in 3/20 (15%), hip in 2/20 (10%), knee in 3/20 (15%), ankle and foot in 7/20 (35%) and thumb in 1/20 (5%) children.

In 15/20 (75%) children MR imaging detected injuries, in 5/20 (25%) children imaging was normal. Table 1 depicts the spectrum of diagnosed injuries in 15 children sorted by anatomic regions.

**Table 1:** Spectrum of injuries in MR imaging of 15 children sorted according to anatomic regions

Case	Region of injury	Anatomical injuries
Nr.15 (Figs. 1 and 2)	thoracic spine	fracture T6 vertebral
Nr. 6	lumbar spine	marrow oedema of sacrum
Nr. 5,12	hip	joint effusion
Nr.10,16,19 (Fig. 3)	knee	fracture of proximal tibia
Nr.1	ankle joint/foot	marrow oedema of distal tibia and talus, fracture of cuboid
Nr.3	ankle joint/foot	fracture of talus, cuboid, ligament rupture
Nr.2,7	ankle joint/foot	ligament rupture
Nr.13 (Figs. 4, 5 and 6)	ankle joint/foot	marrow oedema of talus, cuboid, metatarsals III,IV
Nr.14	ankle joint/foot	fracture of talus, cuboid, navicular, calcaneus
Nr. 4 (Figs. 7 and 8)	ankle joint/foot	marrow oedema of navicular, fracture of first metatarsal
Nr.8	thumb	soft tissue injury

*Note*: In 5/20 (25%) children no injuries were observed

**Fig. 1:**
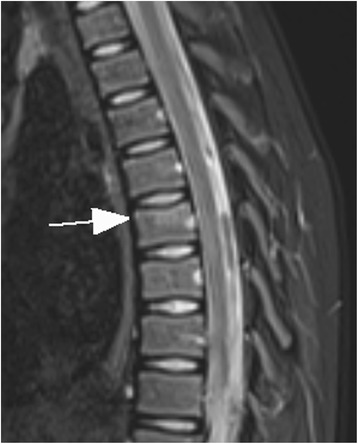
Close up sagittal STIR MR imaging shows oedema and slight impression of the upper endplate of T6 vertebral (*arrow*)

**Fig. 2:**
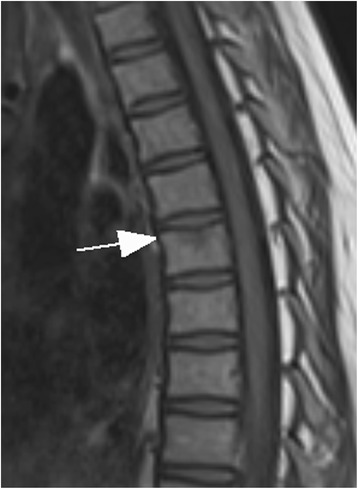
Shows the impression of the upper endplate of T6 vertebral in close up sagittal T1-weighted TSE MR imaging (*arrow*)

**Fig. 3:**
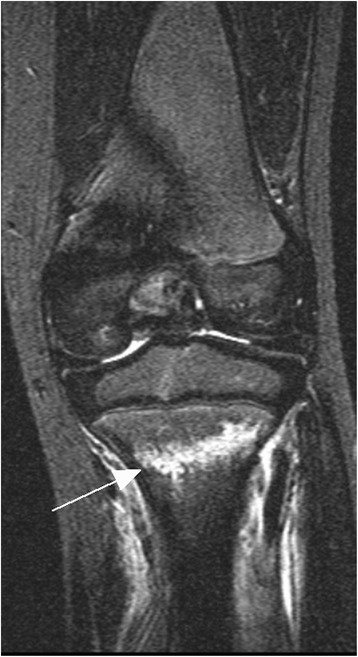
Girl with pain on the tibia after jumping on the trampoline. Coronal STIR sequence shows a fracture (*arrow*) of the proximal tibia metaphysis with surrounding soft tissue damage

**Fig. 4:**
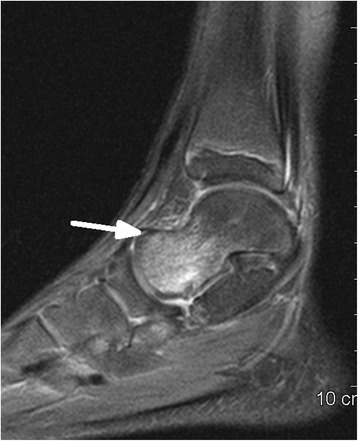
Sagittal PD fat saturated sequence shows marrow oedema of talus (*arrow*) without fracture signs in T1-weighted sequence in Fig. 5

**Fig. 5:**
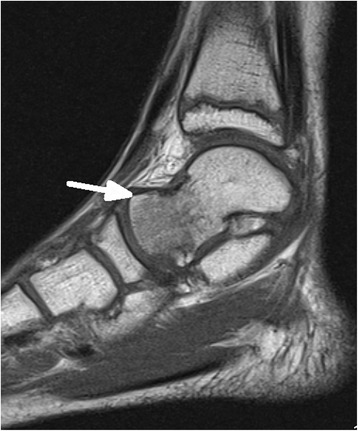
Sagittal T1-weighted sequence did´nt showed a fracture

**Fig. 6:**
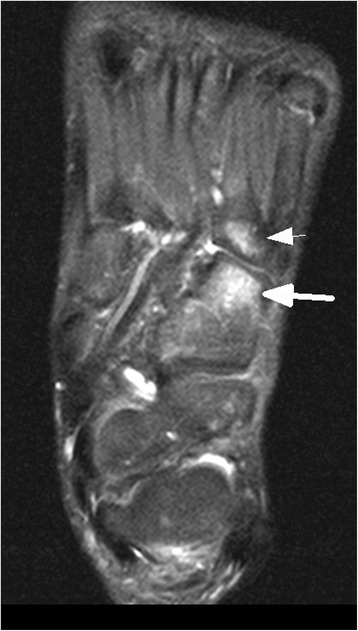
Axial STIR sequence shows marrow oedema of cuboid (*arrow*) and base of metatarsal IV (*arrow ahead*)

**Fig. 7:**
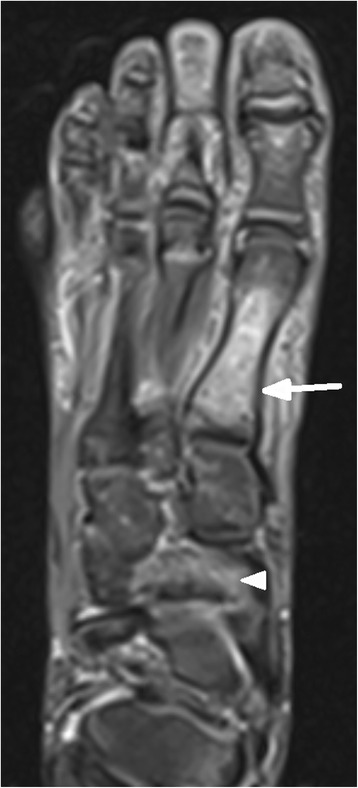
Axial STIR MR imaging shows marrow oedema of navicular (*arrow ahead*) and metatarsal I (*arrow*)

**Fig. 8:**
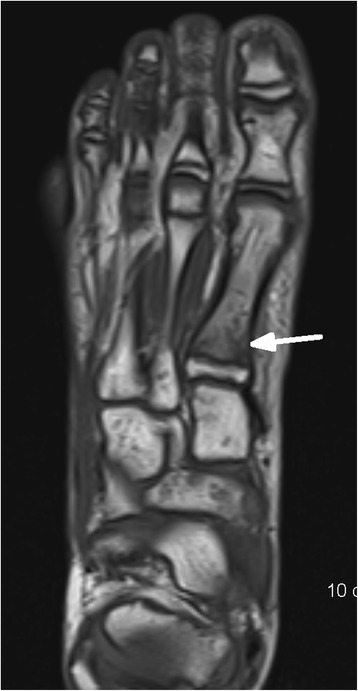
Axial T1-weighted TSE imaging shows a fracture (*arrow*) on the base of the first metatarsal

In 1/15 children a thoracic vertebral fracture was diagnosed (Figs. 1 and 2). Lower extremity injuries were observed in 12/15 (80%) children. Out of these fractures the proximal tibial metaphysis was diagnosed in 3 children (Fig. 3) and injuries of the ankle and foot were diagnosed in 7/15 (47%) patients (Figs. 4, 5, 6, 7 and 8).

In regards to 7 symptomatic children without overt accident we observed fractures of the proximal tibia in 2/7, hip joint effusion in 2/7 and an ankle/foot injury in 1/7 children. The 2/7 remaining children did not present any injuries in MR imaging.

Overall there were no associated spinal cord injuries, no fracture dislocations, no vascular injuries and no head and neck injuries and no surgical management was necessary.

## Discussion

Our retrospective analysis of children examined for complaints related to trampoline use demonstrates positive findings in the majority of scanned children 15/20 (75%) with lower extremity injuries being the predominant type with 12/15 (80%) patients. Our results compare well with some studies where described trampoline injuries most frequently involved the lower extremity [8, 9]. Other studies revealed a preponderance of upper extremity injuries [5, 6, 10]. Klimek et al. [5] for example described as the most common injuries upper extremity fractures such as supracondylar humeral and forearm fractures, which were frequently caused by a direct impact on the ground.

We diagnosed injuries of the ankle and foot in 7/15 (47%) children. In a study performed by Shankar et al. [8] more than 60% of lower extremity injuries involved the ankle and approximately three-quarters of ankle injuries were sprains.

In two studies [2, 4] the trampoline-related fracture of the proximal tibia is the most frequently described trampoline injury. This so called “trampoline fracture” is often observed in children younger than 6 years [2]. We diagnosed this type of fracture in 3/15 children. Due to age-related weakness the proximal tibia is especially vulnerable to axial forces and depression fractures. The injury mechanism was explained in detail by Boyer et al. [2]. According to him, trampoline fractures often occur, when children are using a trampoline with another heavier person. When this person jumps up the trampoline mat recoils upwards from its stretched downward position. If the smaller child lands on the upwards moving mat at the time when its elasticity is reversed by recoil and the springs are shortening to their original length, a significant upward impaction force is applied to the lower extremity of the child. This force may be sufficient to cause fracture. We therefore discourage the trampoline use for children younger than six years of age, especially in regards to jumping simultaneously with older children or adults. The International Trampoline Industry Association and the American Society of Testing and Materials Trampoline Subcommittee issued a revision of performance and safety standards. In this paper printed warnings were included with new trampoline equipment that recommend avoiding somersaulting, restricting multiple jumpers and limiting trampoline use to children 6 years or older [1].

Fractures of the proximal tibial metaphysis may initially appear very subtle on plain radiographs. Therefore in a study of Klimek et al. [5] repeat radiographs after 7–10 days are recommended in children with persistent complaints as an alternative to MRI. Stranzinger et al. [4] described buckle/torus or transverse hairline fractures of the proximal tibial metaphysis as the most common manifestations of the “trampoline fracture”. In their study children with trampoline fractures showed significantly higher anterior tilt angles of the proximal tibial epiphyseal plate on lateral radiographs compared to a control group. They concluded that abnormal anterior tilt angles support the diagnosis of a “trampoline fracture”. They performed follow-up radiographs to exclude valgus deformities and early growth plate closure in their patients. In our study fractures of proximal tibial metaphysis were diagnosed by MRI only, all prior radiographs had been classified as normal.

Our study has some limitations though. Plain X-Rays had been performed at outside, non-pediatric institutions with potential considerable differences in diagnosis. Measurement of the anterior tilt angle was not included in the radiographic analysis. The radiographs were not re-reviewed by pediatric or dedicated musculoskeletal specialists. Another limitation is that our results were not compared with findings in children examined only with radiography, CT or ultrasound. To reduce radiography utilization musculoskeletal sonographic evaluation can be useful to find soft tissue swelling, adjacent and subperiostal hematomas and bone fragments or distal humeral epiphyseal separation [11, 12].

Another limitation of this study is a potential sampling bias. The authors have only knowledge of the children, who were referred for MR imaging. Therefore no broad conclusions regarding the general nature of trampoline-related injuries can be made.

## Conclusions

In the majority of children referred for MR imaging with pain after trampoline MR imaging detects injuries. These injuries are often not visible on plain radiographs. Therefore we recommend a generous use of MR imaging in these children after initial negative plain radiography.

## Abbreviations

MRI: Magnetic resonance imaging
PD: Proton weighted
STIR: Short tau inversion recovery
TSE: Turbo-spin-echo

## References

[1] American Academy of Orthopaedic Surgeons. Trampolines and Trampoline Safety: Position Statement. Rosemont, IL: American Academy of Orthopaedic Surgeons; September, 2010. Available at: www.aaos.org/about/papers/position/1135.asp. Accessed 3 Jan 2012.

[2] Boyer RS, Jaffe RB, Nixon GW, Condon VR (1986). Trampoline fracture of the proximal tibia in children. AJR Am J Roentgenol.

[3] Kakel R (2012). Trampoline fracture of the proximal tibial metaphysis in children may not progress into valgus: a report of seven cases and a brief review. Orthop Traumatol Surg Res.

[4] Stranzinger E, Leidolt L, Eich G, Klimek PM (2014). The anterior tilt angle of the proximal tibia epiphyseal plate: a significant radiological finding in young children with trampoline fractures. Eur J Radiol.

[5] Klimek PM, Juen D, Stranzinger E, Wolf R, Slongo T (2013). Trampoline related injuries in children: risk factors and radiographic findings. World J Pediatr.

[6] Bhangal KK, Neen D, Dodds R (2006). Incidence of trampoline related pediatric fractures in a large district general hospital in the United Kingdom: lessons to be learnt. Inj Prev.

[7] Joeris A, Lutz N, Wicki B, Slongo T, Audigé L (2014). Epidemiological evaluation of pediatric long bone fractures - a retrospective cohort study of 2716 patients from two Swiss tertiary pediatric hospitals. BMC Pediatr.

[8] Shankar A, Williams K, Ryan M (2006). Trampoline-related injury in children. Pediatr Emerg Care.

[9] Königshausen M, Gothner M, Kruppa C, Dudda M, Godry H, Schildhauer TA, Seybold D (2014). Trampoline-related injuries in children: an increasing problem. Sportverletz Sportschaden.

[10] Black GB, Amadeo R (2003). Orthopedic injuries associated with backyard trampoline use in children. Can J Surg.

[11] Saul T, Ng L, Lewiss RE (2013). Point-of-care ultrasound in the diagnosis of upper extremity fracture-dislocation. A pictorial essay. Med Ultrason.

[12] Supakul N, Hicks RA, Caltoum CB, Karmazyn B (2015). Distal humeral epiphyseal separation in young children: an often-missed fracture-radiographic signs and ultrasound confirmatory diagnosis. AJR Am J Roentgenol.

